# Clinical and Biochemical Predictors of Nonalcoholic Fatty Liver Disease among Type 2 Diabetes Mellitus Patients at Primary Health Care Level in South Western Saudi Arabia

**DOI:** 10.3390/diagnostics10100809

**Published:** 2020-10-12

**Authors:** Suliman M. Al Humayed, Abdullah A. Al Sabaani, Ahmed A. Mahfouz, Nabil J. Awadalla, Mustafa Jafar Musa, Ayyub Patel

**Affiliations:** 1 Department of Internal Medicine, College of Medicine, King Khalid University, Abha 61421, Saudi Arabia; s_humayed@yahoo.com; 2 Department of Family and Community Medicine, College of Medicine, King Khalid University, Abha 61421, Saudi Arabia; dr.alsabaani@hotmail.com (A.A.A.S.); njgirgis@yahoo.co.uk (N.J.A.); 3 Department of Epidemiology, High Institute of Public Health, Alexandria University, Alexandria 21511, Egypt; 4 Department of Community Medicine, College of Medicine Mansoura University, Mansoura 35516, Egypt; 5 Department of Applied Radiologic Technology, College of Applied Medical Sciences, University of Jeddah, Jeddah 21959, Saudi Arabia; musa30000@yahoo.com; 6 Department of Clinical Biochemistry, College of Medicine, King Khalid University, Abha 61421, Saudi Arabia; ayyub@kku.edu.sa

**Keywords:** nonalcoholic fatty liver disease, type 2 diabetes mellitus, ROC analysis, ALT, Saudi Arabia

## Abstract

Objectives: To predict the role of different clinical and biochemical parameters in identifying nonalcoholic fatty liver disease (NAFLD) among patients with type 2 diabetes mellitus (T2DM) in Abha city, southwestern Saudi Arabia. Methods: A stratified random sample was selected. A detailed clinical and biochemical examinations were performed. Using portable abdominal ultrasound examination, NAFLD was identified. The study used receiver operating characteristic (ROC) analysis. Results: The study covered 237 T2DM patients. NAFLD was detected among 174 patients. Area under the curve (AUC) calculations showed that the ability of age, duration of DM in years, and body mass index to predict NAFLD was poor (AUC < 0.6). Similarly, biochemical factors like HbA1c%, AST, cholesterol, triglycerides, HDL, LDL, and VLDL were poor in discriminating between those with and without NAFLD among T2DM. On the other hand, the ability of ALT to predict NAFLD among T2DM was good (AUC = 0.701, 95% CI: 0.637–0.761). The analysis identified the optimal cutoff point of ALT to be ≤22.1 nmol/L. The corresponding sensitivity was 60.7% (95% CI: 53.0–68.0) and specificity was 62.5% (95% CI: 49.5–74.3). Conclusions: Early identification of NAFLD among T2DM is important. A threshold cutoff value of 22.1 nmol/L of ALT has been identified to predict NAFLD. They should be referred for ultrasound examination for NAFLD.

## 1. Introduction

Nonalcoholic fatty liver disease (NAFLD) is described as hepatic steatosis not produced by extra consumption of alcohol, viruses such as hepatitis B or C, autoimmune hepatitis, using of hepatotoxic drugs or other compounds, or rare genetic forms [1]. It includes a variety of illnesses from simple steatosis to nonalcoholic steatohepatitis (NASH) and cirrhosis. NAFLD is at present the most widespread liver illness with a predictable global occurrence of 25% [2]. Based on the technique of identification, 65–87% of patients with type 2 diabetes (T2DM) have NAFLD [3]. The relationship between NAFLD and type 2 diabetes mellitus (T2DM) has been well proven, which could be described by the insulin challenge and compensatory hyperinsulinemia advancing to faulty lipid metabolism and hepatic triglyceride (TG) increase in NAFLD or to b-cell malfunction in T2DM [4]. Studies revealed that NAFLD–T2DM correlation is in both directions [5].

A systematic review published in 2016 [6] estimated the occurrence of type 2 diabetes mellitus in Saudi Arabia to be 32.8%. However, the article expected the occurrence to be 35.37% in 2020; 40.37% in 2025, and 45.36% in the year 2030. Another systematic review in 2017 [7] reported the same trend of overall increasing prevalence of T2DM in Saudi Arabia. Study in Jazan region, Saudi Arabia, reported a prevalence of NAFLD of 47.8% (95% CI 41.1–54.6) among T2DM [8]. With the predicted increase of the occurrence of T2DM, the occurrence of NAFLD is anticipated to increase. Globally, duo to epidemics of DM in industrialized countries, it is expected also to bring about an impressive increase in the occurrence of NAFLD in these regions [9]. 

Ultrasonography is harmless and worthwhile for detecting NAFLD among T2DM patients as compared to histological diagnosis (liver biopsy) [10]. Ultrasound services are not usually available at primary health care (PHC) level in Abha city, southwestern Saudi Arabia. Patients are usually referred to a secondary level for this service. In spite of the elevated occurrence and severe medical consequences of NAFLD in cases with T2DM, it is commonly unnoticed in clinical practice. Improving understanding about the status of NAFLD in cases with T2DM among principal interested party (primary care doctors and consultants) should be highlighted.

Data regarding the predicting values of clinical and biochemical laboratory tests at primary health care level in predicting NAFLD are deficient. The main objective of this research was to use receiver operating characteristic (ROC) analysis to predict the role of different clinical and biochemical parameters in identifying NAFLD among patients with T2DM in Abha city, southwestern Saudi Arabia.

## 2. Materials and Methods

### 2.1. Study Design and Patients

The design was cross-sectional. The study targeted patients going to primary health care centers diabetes clinics at Abha city. The principal inclusion criteria were adult patients (18 years and above), with T2DM, while patients with concomitant liver disease (HBV or HCV known cases) and individuals who drink alcohol or taking steatogenic medications were not included.

### 2.2. Sample Size and Sampling

"MedCalc" statistical software version 16.4.3 (MedCalc Software bv, Ostend, Belgium; https://www.medcalc.org; 2016) [11] was used for calculating the minimal sample size needed for area under the curve calculation (AUC) analysis. To have an area under the curve of 0.70 for a particular variable to be significantly different from the null hypothesis value of 0.5 (denoting no discriminating influence), at alpha level of 0.01 and beta level of 0.01 at a null hypothesis value of 0.5 (no discriminating power), a needed sample of 182 participants was calculated [12]. 

The sample units were selected using stratified random sample technique. The sample was selected from the study ten primary health care centers (PHCCs) taking into consideration the relative catchment population in each center (to avoid clustering effect). 

### 2.3. Sociodemographic, Clinical, and Biochemical Data

Patients were interviewed and their records were reviewed to gather information regarding age, gender, and duration of diabetes. Weight and standing height were assessed. Body mass index (BMI) was computed as weight (kg)/height (m^2^). 

Ten milliliters of peripheral blood were taken from accepting patients and transferred to the laboratory directly. A benchtop centrifuge (5000 rpm, 5 min, Eppendorf 5810, Hamburg, Germany) was used to separate the blood into plasma, serum, RBCs, and buffy coat. The separated components were kept in −80 °C freezers (Revco Scientific Deep Freezer, USA). All samples were split up into plasma, serum, red blood cells, and buffy coat, and stored in −80 °C freezers. Plasma concentrations of ALT, AST, total cholesterol, triglycerides, high density lipoprotein, low density lipoprotein, and very low-density lipoprotein were evaluated using a chemistry analyzer (AU5800 Analyzer, Beckman Coulter, Brea, CA, USA). HbA1c levels were evaluated by HPLC method using Bio-Rad Variant II System (Bio-Rad Laboratories) in red blood cells [13]. Calibration of the devices were done according to the standard methods.

### 2.4. Abdominal Ultrasound Examination

The participants were requested to fast for 6 h prior to the examination. The ultrasound was done with the participant lying horizontally with the face and torso facing up. Relevant gel was smeared to the abdomen. Longitudinal and transverse imaging was made. The consistency and dimension of the liver were recorded. The examination was done by one skilled radiologist using a sensitive ultrasound machine (LOGIC Book XP; General Electric, Jiangsu, China) with a 3.5 MHZ convex transducer (GE 8 L-RS ultrasound probe). Calibration of the device was performed prior to each clinical session at the PHCC. The normal liver parenchyma has homogenous echo texture with echogenicity equal or slightly greater than that of the renal cortex and spleen. Diagnosis of fatty liver by ultrasonography is defined by the presence of at least two or three abnormal findings including diffusely increased echogenicity (“bright”) liver with liver echogenicity stronger than kidney or spleen and either deep attenuation of ultrasound signal or vascular blurring of the fatty liver showing higher echogenicity than the renal cortex in both sides. 

### 2.5. Statistical Analysis

Data were reviewed, refined, and analyzed using the “SPSS” software package, version 22 (IBM Corp. Released 2013. IBM SPSS Statistics for Windows, Version 22.0. Armonk, NY: IBM Corp.) and “MedCalc” statistical software version 16.4.3 (MedCalc Software bv, Ostend, Belgium; https://www.medcalc.org; 2016). Arithmetic mean, 95% confidence interval of the mean and standard error of the mean were used to present clinical and biochemical data. When comparing the 95% CI among those with NAFLD and those without, statistically significant differences were taken into account if the two confidence intervals did not overlap. 

A receiver operating characteristic (ROC) curve was created using “MedCalc” software to predict the role of different clinical and laboratory parameters in identifying T2DM patients with NAFLD. It showed the performance of the cutoff points in terms of sensitivity versus 1-specificity. The area under the curve (AUC) is an estimate of the accurateness of cutoff point. The AUC estimate lies between 0.5 and 1. Values <0.7 indicate a weak classifier and 1 indicates an outstanding classifier.

### 2.6. Ethical Approval

The research was approved by the Research Ethics Committee, College of Medicine, King Khalid University (HA-O7-B-012, dated 22 June 2016). Written informed consents were collected from participants. Approval was also obtained from the Aseer Directorate of Health. 

## 3. Results

The present study included 237 T2DM patients (157 males and 80 females). Their age ranged from 20 to 100 years, with an average of 56.5 and a median of 51 years. Most of them were married (82%) and nonsmokers (82%). The average duration of DM was 10.8 years. 

NAFLD was detected among 174 T2DM. Table 1 shows the arithmetic mean, 95% confidence intervals of the mean, and standard error of the mean of clinical and biochemical variables among T2DM patients with and without NAFLD in Abha city. The average age of T2DM with NAFLD was 55.9 years (95% CI: 53.2–58.5) and those without NAFLD was 57.1 years (95% CI: 55.9–59.1). The overlap in 95% CIs among both groups indicated nonsignificant differences regarding age. Similarly, BMI and duration of diabetes were not significantly different between both groups. 

The average HbA1c of T2DM with NAFLD was 8.7% (95% CI: 8.4–9.1) and those without NAFLD was 8.7 (95% CI: 7.6–9.4). The overlap in 95% CIs among both groups indicated nonsignificant differences regarding HbA1c. Similarly, AST, cholesterol, triglycerides, HDL, LDL, and VLDL were not significantly different between both groups. On the other hand, the average ALT of T2DM with NAFLD was 25.8 (95% CI: 24.7–26.9) and those without NAFLD was 22.5 (95% CI: 21.1–23.8). The lack of overlap in 95% CIs among both groups indicated a significant difference regarding ALT.

Table 2 shows ROC curve analysis of the clinical and biochemical predictors of NAFLD among T2DM in Abha city. The ability of age in years expressed as a continuous variable, to discriminate between those with and without NAFLD was poor (AUC < 0.7). Similarly, other clinical factors like BMI and duration of DM in years were poor in discriminating between those with and without NAFLD among T2DM. The corresponding AUC values were 0.457, 0.5950, and 0.592, respectively.

**Figure 1:**
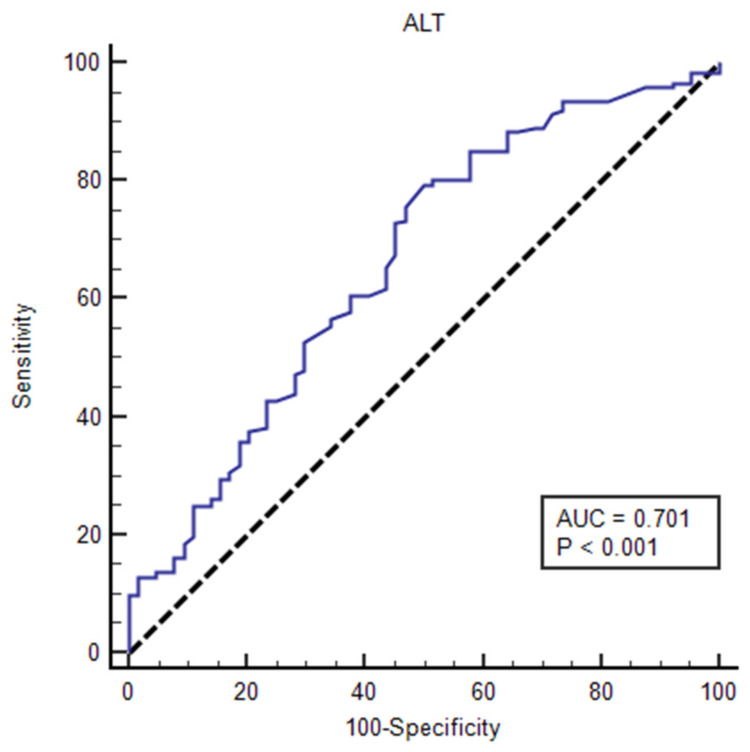
Receiver operating characteristic curve analysis of alanine aminotransferase (ALT) in IU/L to predict nonalcoholic fatty liver disease (NAFLD) among study sample of type 2 diabetes (T2DM) at Abha city.

The ability of HbA1c% expressed as a continuous variable, to discriminate between those with and without NAFLD was poor (AUC < 0.7). Similarly, other biochemical factors like AST, cholesterol, triglycerides, HDL, LDL, and VLDL were poor in discriminating between those with and without NAFLD among T2DM. On the other hand, the ability of ALT, expressed as a continuous variable, to predict NAFLD among T2DM was good (AUC = 0.701, 95% CI: 0.637–0.761). The ROC curve (Table 3 and Figure 1) identified the optimal cutoff point of ALT to be ≤22.1 nmol/L. The corresponding sensitivity was 60.7% (95% CI: 53.0–68.0) and the specificity was 62.5% (95% CI: 49.5–74.3).

## 4. Discussion

Type 2 diabetes (T2DM) is a compound metabolic condition that has turn out to be a most important public health concern globally and, in particular, in Saudi Arabia [6]. T2DM can lead to damage to the pancreas, insulin system, and the liver. A latest meta-analysis discovered that the worldwide occurrence of NAFLD among patients with T2DM is about 55% [14]. Overall, as patients with T2DM are affected by a greater occurrence of NAFLD, there must be systematic examination for NAFLD to foster early discovery and avoid complications [15]. The gold standard for the identification and classification of NAFLD is liver biopsy. It is an invasive procedure, frequently associated with distress and discomfort. Ultrasound is the suggested first-line examination technique for patients with T2DM by the European NAFLD guidelines [1]. However, there are other different imaging techniques used for noninvasive assessment of NAFLD. These include, hepatorenal ration, acoustic structure quantification, controlled attenuation parameter, magnetic resonance imaging, and CT-scan. These techniques are more accurate than conventional ultrasound, but less commonly used in clinical and population-based research settings [16]. Yet, there are some drawbacks of ultrasonography use, comprising observer reliance, subjective assessment, and low ability to measure the extent of fatty infiltration, which have addressed concerns [17]. A meta-analysis study was conducted to evaluate the validity and reliability of ultrasound to identify fatty liver, compared to biopsy, the gold-standard test. It showed that the overall sensitivity of ultrasound to identify moderate to severe biopsy diagnosed fatty liver from the lack of steatosis was 84.8% and the AUC was 0.93. Moreover, ultrasounds have accuracy for the diagnosis of more than or equal to 10% of steatosis between 0.91 and 0.93, and specificity ranged between 88% and 99%. However, the diagnostic accuracy of ultrasound may be reduced by the concomitant chronic hepatic conditions and obesity. Another disadvantage of ultrasound was the reported wide range of intra-observer kappa values ranged between 0.54 and 0.92 and interobserver values ranged between 0.44 and 1.00 [18]. In the present study, only one examiner did the ultrasound examination to avoid the possibility of interobserver disagreement.

Ultrasound services are not normally available at primary health care (PHC) level in Abha city. In the present study, researchers were evaluating and modeling common clinical and biochemical investigations available at PHC level one by one, with the goal of establishing a scientific risk prediction model appropriate for family physicians to perform early investigation of NAFLD among patients with diabetes.

The present study documented the poor discriminating ability of age in years and other clinical factors like BMI and duration of DM in years in distinguishing between those with and without NAFLD among T2DM at primary health care level in Abha city. Similar results were reported among Iraqi patients [19] and in Jazan, Saudi Arabia [8]. On the other hand, another study in Bucharest, Romania, identified BMI as clinical predictor of NAFLD [20]. Recently, researchers developed a sensitive index, including clinical (age, sex, and BMI) and biochemical data and Dallas steatosis index, to predict NAFLD [21]. Similarly, other scoring systems were developed [22]. Yet, no single clinical factor was identified as a predictor for NAFLD.

The present study showed that the ability of HbA1c% to distinguish between those with and without NAFLD was poor (AUC < 0.6). Our results suggest that HbA1c level is inappropriate predictor of NAFLD among T2DM patients. Similar findings were described in preceding studies [23,24]. On the other hand, a tendency of raised HbA1c level among NAFLD was reported among NAFLD diabetic patients in another reports [25,26]. The reason of this disagreement could be elucidated by the variation in the level of HbA1c that might happen from time to time. 

In the present study, blood lipids like cholesterol, triglycerides, HDL, LDL, and VLDL were poor indicator in discriminating between those with and without NAFLD among T2DM. On the other hand, a study in Serbia [27], using ROC analysis, found that the addition of fatty liver risk factors (e.g., age, gender, body height, smoking status, diabetes duration, and drugs metabolized in liver) to each analyzed biochemical blood lipids, augmented the ability to distinguish T2DM patients with and without NAFLD. Another study in China [28], used an index, LAP (lipid accumulation product) composed of waist circumference and triglycerides level. Using ROC analysis, the researchers found that LAP exhibited high diagnostic accuracy for identifying NAFLD. 

Regarding liver enzymes, AST and ALT, the present study showed that AST was poor in discriminating between those with and without NAFLD among T2DM. On the other hand, the ability of ALT, expressed as a continuous variable, to predict NAFLD among T2DM was good. The ROC curve analysis identified the optimal cutoff point of ALT to be 22.1 nmol/L. The corresponding sensitivity was 60.7% (95% CI: 53.0–68.0) and the specificity was 62.5% (95% CI: 49.5–74.3).

Alanine aminotransferase (ALT) enzyme activity is mainly found in the liver, but its activity, although much lower, is also found in many other tissues including muscle, heart, kidney, brain, and adipose tissue. Prospective studies have revealed that raising liver enzyme levels, chiefly ALT, predicts incident diabetes and likely reveals NAFLD [29]. Researchers recommended a lowered ALT cut-point for women (ALT< 19 U/L) to increase sensitivity for NAFLD and chronic liver disease [30].

Similar to our study, a published article in Nepal found the area under the receiver operating characteristic curve for the prediction of fatty liver based solely on the ALT to be 0.84 with a confidence interval (CI) between 0.76 and 0.92 (*p* < 0.05) [31]. Similarly, a study in Belgrade among Montenegrin population found that ALT was independent predictor for NAFLD. They found a lower cutoff point associated for NAFLD of 22 IU/L (AUC = 0.804, sensitivity 61%, and specificity of 95%) [32].

A study in the United Kingdom demonstrated that NAFLD identified by ultrasonography was the most frequent reason of abnormal liver biochemistry. Diabetic patients with NAFLD had significantly greater ALT level in contrast to diabetic subjects without NAFLD [33].

Our study was unique in describing the predictors connected to NAFLD among T2DM patients in Abha city, southwestern Saudi Arabia. However, certain limitations are existing. One of the limitations of the study was the use of ultrasonography as a proxy of NAFLD. The gold standard for the identification and classification of NAFLD is liver biopsy. Generalization of our findings should be made with some concern as the study comprised only T2DM frequently attending PHCCs in Abha city. Similarly, the relatively small sample size of the study should be taken into consideration. Another limitation was related to the nature of the study being cross-sectional. Identification of the biomarkers and NAFLD were measured at the same point of time.

## 5. Conclusions

In spite of the high occurrence and important medical consequences of NAFLD in patients with T2DM, it is usually ignored in clinical practice. Primary care practitioners are at the frontline of potentially facing patients with NAFLD. T2DM cases with ALT values of 22.1 nmol/L and more should be referred for ultrasound examination for the possibility of NAFLD.

## Figures and Tables

**Table 1:** Arithmetic mean, 95% confidence intervals of the mean, and standard error of the mean of clinical and biochemical variables among type 2 diabetes (T2DM) patients (237) with and without nonalcoholic fatty liver disease (NAFLD) in Abha city.

Variable	NAFLD by Ultrasound			
	Positive (174)		Negative (63)	
	Mean (95% CI)	SE	Mean (95% CI)	SE
Clinical				
Age in years	55.9 (53.2–58.5)	1.32	57.1 (55.9–59.1)	0.86
BMI	29.9 (29.0–30.8)	0.41	27.9 (26.6–29.1)	0.59
Duration of DM in years	9.3 (8.2–10.5)	5.57	12.2 (9.9–14.6)	0.78
Biochemical				
HbA1c %	8.7 (8.4–9.1)	0.15	8.7 (7.6–9.4)	0.27
ALT in IU/L	25.8 (24.7–26.9)	0.54	22.5 (21.1–23.8)	0.68
AST in IU/L	20.2 (17.8–22.7)	1.25	17.1 (15.9–18.3)	0.59
Cholesterol in mg/dL	146.4 (138.8–153.9)	3.81	151.7 (140.3–163.2)	5.74
Triglycerides in mg/dL	182.4 (170.8–194.6)	5.91	175.2 (154.1–196.2)	10.51
HDL in mg/dL	28.4 (27.3–29.5)	0.55	29.7 (27.9–31.5)	0.88
LDL in mg/dL	84.1 (77.3–90.8)	3.4	86.9 (75.0–98.9)	5.91
VLDL in mg/dL	36.4 (34.1–38.8)	1.17	35.1 (30.8–39.2)	2.10

95% CI: 95% confidence interval; SE: standard error of the mean; BMI: body mass index; HbA1c: glycated hemoglobin; ALT: Alanine aminotransferase; AST: Aspartate aminotransferase; HDL: high-density lipoproteins; LDL: low-density lipoproteins; VLDL: Very-low-density lipoproteins.

**Table 2:** Clinical and biochemical predictors of NAFLD among T2DM in Abha city.

Variable	AUC	SE	95% CI	*P*
Clinical				
Age in years	0.457	0.044	0.371–0.543	0.308
BMI	0.595	0.039	0.530–0.657	0.017
Duration of DM in years	0.592	0.041	0.528–0.654	0.021
Biochemical				
HbA1c %	0.504	0.043	0.440–0.568	0.921
ALT in IU/L	0.701	0.040	0.637–0.761	0.001
AST in IU/L	0.627	0.039	0.562–0.689	0.001
Cholesterol in mg/dL	0.510	0.042	0.445–0.575	0.817
Triglycerides in mg/dL	0.537	0.043	0.471–0.601	0.401
HDL in mg/dL	0.552	0.041	0.486–0.616	0.215
LDL in mg/dL	0.509	0.044	0.422–0.595	0.839
VLDL in mg/dL	0.537	0.043	0.452–0.622	0.386

AUC: area under the curve, 95% CI: 95% confidence interval; SE: standard error; BMI: body mass index; HbA1c: glycated hemoglobin; ALT: Alanine aminotransferase; AST: Aspartate aminotransferase; HDL: high-density lipoproteins; LDL: low-density lipoproteins; VLDL: Very-low-density lipoproteins.

**Table 3:** Results of receiver operating characteristics for alanine aminotransferase (ALT) to predict NAFLD among T2DM in Abha city.

Condition	AUC (95% CI)	Optimal Cutoff Point of ALT (IU/L)	Sensitivity % (95% CI)	Specificity % (95% CI)
NAFLD	0.701 (0.637–0.761)	≤ 22.1	60.7 (53.0–68.0)	62.5 (49.5–74.3)

AUC: area under the curve, 95% CI: 95% confidence interval; NAFLD: nonalcoholic fatty liver disease; ALT: Alanine aminotransferase.
